# A Fatty Acid Glycoside from a Marine-Derived Fungus Isolated from Mangrove Plant *Scyphiphora hydrophyllacea*

**DOI:** 10.3390/md10030598

**Published:** 2012-03-06

**Authors:** Yan-Bo Zeng, Hui Wang, Wen-Jian Zuo, Bo Zheng, Tao Yang, Hao-Fu Dai, Wen-Li Mei

**Affiliations:** 1 Hainan Key Laboratory for Research and Development of Natural Products from Li Folk Medicine, Institute of Tropical Bioscience and Biotechnology, Chinese Academy of Tropical Agricultural Sciences, Haikou 571101, Hainan, China; Email: zengyanbo@163.com (Y.-B.Z.); wanghuilily2000@163.com (H.W.); zuowenjian88@163.com (W.-J.Z.); zhengbofootball@163.com (B.Z.); yangtaoyy3@126.com (T.Y.); 2 Haikou Key Laboratory for Research and Development of Tropical Natural Products, Haikou 571101, Hainan, China

**Keywords:** marine endophyte, secondary metabolite, *Scyphiphora hydrophyllacea*, *R*-3-hydroxyundecanoic acid methylester-3-*O*-α-l-rhamnopyranoside

## Abstract

To study the antimicrobial components from the endophytic fungus A1 of mangrove plant *Scyphiphora hydrophyllacea* Gaertn. F*.*, a new fatty acid glucoside was isolated by column chromatography from the broth of A1, and its structure was identified as *R*-3-hydroxyundecanoic acid methylester-3-*O*-α-l-rhamnopyranoside (**1**) by spectroscopic methods including 1D and 2D NMR (HMQC, ^1^H-^1^H COSY and HMBC) and chemical methods. Antimicrobial assay showed compound **1** possessed modest inhibitory effect on *Saphylococcus aureus *and methicillin-resistant *S*. *aureus* (MRSA) using the filter paper disc agar diffusion method.

## 1. Introduction

**Figure 1:**
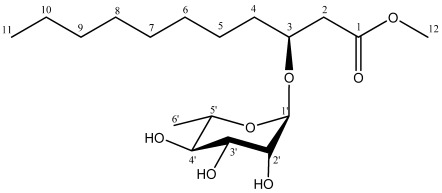
The structure of compound **1**.

Marine-derived fungi have proved to be a rich source of structurally unique and biologically active secondary metabolites [1]. Marine natural products have been the focus of discovery for new products of chemical and pharmacological interest. Over the last decade, many new metabolites have been isolated and identified from marine-derived fungi and their biological activities have been evaluated [2,3]. Previously, phytochemical studies showed that mangrove plant *Scyphiphora hydrophyllacea* Gaertn. F. is a rich source of new iridoids, which have been found to possess cytotoxic activity [4,5,6,7]. Therefore, endophytic fungi isolated from *S. hydrophyllacea* were studied [8]. During our ongoing investigation on new natural antibacterial agents from endophytic fungi isolated from mangrove plant, the fungus A1 isolated from *S. hydrophyllacea* attracted our attention because the EtOAc extract of the fungus culture showed inhibitory activity against methicillin-resistant *Saphylococcus aureus* (MRSA). Further investigation on the secondary metabolites from the culture broth of the fungus A1 led to the isolation of a new fatty acid glycoside by using column chromatography, and its structure was unambiguously elucidated as *R*-3-hydroxyundecanoic acid methylester-3-*O*-α-l-rhamnopyranoside (**1**) (Figure 1). Herein we report the isolation, structural elucidation, and biological activity of this compound.

## 2. Results and Discussion

### 2.1. Structural Elucidation

**Figure 2:**
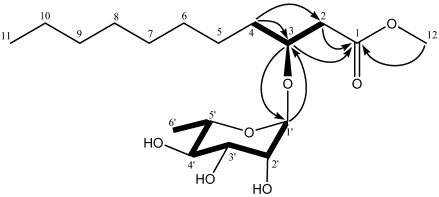
Key ^1^H-^1^H COSY (bold lines) and HMBC (arrows) correlations of compound **1**.

Compound **1 **was obtained as colorless oil. The IR spectrum of **1** showed absorption bands for OH (3404 cm^−1^), C=O (1711 cm^−1^), and C–O (1050 cm^−1^) functional groups in the molecule. The [M + Na]^+^ at *m*/*z* 385.2201 in the HR-ESI-MS spectrum corresponds to the molecular formula C_18_H_34_O_7_ (calc. 385.2202), indicating two degrees of unsaturation. The ^13^C-NMR (Table 1) along with the DEPT experiments revealed the presence of 18 carbon atoms including one carbonyl carbon, six methines, eight methylenes, and three methyl groups. Five carbon signals (δ_C_ 98.1, 72.9, 71.6, 71.4, 68.4) resonated in the region 60–100 ppm and a methyl group (δ_C_ 17.3) were ascribed to be a rhamnose unit. The anomeric proton of rhamnose at 4.87 (1H, *d*, *J* = 1.2 Hz) suggested that the rhamnose was in α-orientation. The HMQC spectrum allowed all carbon resonances to be unambiguously assigned to the resonances of their directly attached protons. In the ^1^H-^1^H COSY spectrum, the correlations of H-1′ to H-2′, H-2′ to H-3′, H-3′ to H-4′, H-4′ to H-5′ and H-5′ to H-6′ allowed to structure the rhamnose. In the HMBC spectrum, the correlation between H-1′ and C-3 (δ_C_ 74.0) suggested the rhamnose unit link at the C-3 position. With a shift of 172.2 ppm, the carbonyl carbon C-1 was determined to be a conjugated ester function. The chemical shift of C-12 (δ_C_ 51.7) and the HMBC correlation from H-12 (δ_H_ 3.77) to C-1 (δ_C_ 172.2) revealed that C-12 was connected with C-1 through an oxygen atom (Figure 2). The HMBC correlations of H-2 to C-1, H-2 to C-3, H-3 to C-1 and ^1^H-^1^H COSY correlation of H-2 to H-3 suggested that C-1 was connected with C-3 via C-2, as shown in Figure 2. The ^1^H-^1^H COSY correlations of H-3 to H-4 and the HMBC correlations of H-4 to C-3, H-4 to C-2 revealed that C-4 was connected with C-3. The remaining fatty acid chain was assigned by comparing with the spectral data of *R*-3-hydroxydodecanoic acid methylester [9]. Compared to known compound rhamnolipid 3, compound **1** had one more methylene in fatty acid chain and the C-1 hydroxyl was methylated [10]. Thus, the primary structure of **1** was obtained (Figure 1). The relative configuration of C1′-C5′ centers of the sugar moiety could be deduced by analysis vicinal coupling constants for the sugar proton signals. Thus, a H1′/H2′ diequatorial relationship was indicated by the 1.2 Hz coupling constant observed at H1. Likewise, the big coupling constant observed in the triplet signal for H4′ (δ 3.44, *t*, *J* = 9.6 Hz) demonstrated that H3′, H4′, and H5′ were axial. The stereochemistry of the asymmetric center of **1** at C-3 was proposed to be *R *by acid hydrolysis, similar to *R*-3-hydroxydecanoic acid methylester [9], based on the negative sign of its specific rotation ([α]^21^_D_ −20.5, (*c* 1.0, CHCl_3_)). Meanwhile, the sugar moiety of **1** was determined as L-rhamnose based on the positive sign of its specific rotation [11]. Thus, the structure of **1** was established as *R*-3-hydroxyundecanoic acid methylester-3-*O*-α-l-rhamnopyranoside.

**Table 1:** ^1^H- and ^13^C-NMR data of compound **1**.

No.	δ_C_	δ_H_
1	172.2 (*s*)	
2	40.1 (*t*)	2.51 (2H, *m*)
3	74.0 (*d*)	4.08 (1H, *m*)
4	33.2 (*t*)	1.52 (2H, *m*)
5	24.8 (*t*)	1.25–1.28 (2H, overlapped)
6	29.2 (*t*)	1.25–1.28 (2H, overlapped)
7	29.7 (*t*)	1.25–1.28 (2H, overlapped)
8	29.6 (*t*)	1.25–1.28 (2H, overlapped)
9	31.8 (*t*)	1.25–1.28 (2H, overlapped)
10	22.6 (*t*)	1.25–1.28 (2H, overlapped)
11	14.0 (*q*)	0.88 (3H, *t,* 6.8 Hz)
12	51.8 (*q*)	3.68 (3H, *s*)
1′	98.1 (*d*)	4.87 (1H, *d*, 1.2 Hz)
2′	71.4 (*d*)	3.86 (1H, *brs*)
3′	71.6 (*d*)	3.69 (1H, overlapped)
4′	72.9 (*d*)	3.44 (1H, *t*, 9.6 Hz)
5′	68.4 (*d*)	3.60 (1H, *m*)
6′	17.3 (*q*)	1.25–1.28 (3H, overlapped)

### 2.2. Biological Activities

Antibacterial tests demonstrated that compound **1** showed modest inhibitory effects on *Staphylococcus aureus* and methicillin-resistant *S*. *aureus* (MRSA), diameters of inhibition zones of which were 9.8 and 10.7 mm, respectively. The diameters of inhibition zones of the positive control, kanamycin sulfate, were 22.8 and 18.2 mm, respectively.

## 3. Experimental Section

### 3.1. General

The IR spectrum was obtained on a Nicolet 380 FT-IR instrument from KBr pellets. The UV spectrum was measured on a Shimadzu UV-2550 spectrometer. Optical rotation was recorded using a Rudolph Autopol III polarimeter (USA). The NMR spectra were recorded on a Bruker AV-400 spectrometer, using TMS as an internal standard. The HR-ESI-MS spectrum was measured with an API QSTAR Pulsar mass spectrometer. Column chromatography (CC) was performed with silica gel (Marine Chemical Industry Factory, Qingdao, China) and Sephadex LH-20 (Merck, Darmstadt, Germany). TLC was preformed with silica gel GF254 (Marine Chemical Industry Factory, Qingdao, China). 

### 3.2. Fungal Material and Fermentation

The marine-derived fungus A1 (unidentified) was isolated from the leaves of mangrove plant *Scyphiphora hydrophyllacea* Gaertn. F. (No. SH20081205), which were collected in Wenchang county in Hainan Province (China) in December, 2008. The strain was deposited in the Institute of Tropical Bioscience and Biotechnology, Chinese Academy of Tropical Agricultural Sciences, Haikou, China, and maintained on potato dextrose agar (PDA) slant at 4 °C. The marine-derived fungus A1 was grown on PDA at room temperature for 5 days. Three pieces of mycelial agar plugs (0.5 × 0.5 cm^2^) were inoculated into 1 L Erlenmeyer flasks containing 300 mL potato dextrose broth. The cultivation was shaken at 120 rpm at room temperature for 7 days, and then kept in still at room temperature for 45 days.

### 3.3. Extraction and Isolation

The culture broth (130 L) was filtered to give the filtrate and mycelia. The filtrate was evaporated *in vacuo* to small volume and then partitioned in succession between H_2_O and petroleum ether, EtOAc, *n*-Butanol. The EtOAc solution was evaporated under reduced pressure to give a crude extract (10.5 g), which was separated into 11 fractions (Fr.1–Fr.11) on a silica-gel column using a step gradient elution of CHCl_3_/MeOH (1:0 → 0:1). Fraction 10 (427.1 mg) was purified by column chromatography over Sephadex LH-20 with CHCl_3_/MeOH (1:1) as eluent, yielding 3 subfractions. Subfraction 1 (90.8 mg) was submitted to chromatography on a silica-gel column with petroleum ether/acetone (3:1) as eluent, yielding compound **1** (14.5 mg).

### 3.4. Acid Hydrolysis

Compound **1** (5 mg) was dissolved in MeOH (2 mL) and 5% H_2_SO_4_ solution (2 mL) and hydrolyzed under refluxed at 80 °C for 3 h. The mixture was diluted twofold with distilled water, and partitioned between CHCl_3_ and H_2_O. The aqueous layer was neutralized with aqueous NaHCO_3_ solution (1 M) and evaporated to afford the residue. The TLC analysis (CHCl_3_–MeOH 8:2, v/v, *R*_f_ = 0.43) with authentic sample suggested the presence of L-rhamnose, and its identity was confirmed after preparative TLC in the same solvent and by measurement of its optical rotation dispersion as L-rhamnose ([α]^21^_D_ +7.7, (*c* 0.15, H_2_O)). The CHCl_3_ extract was subjected to CC (silica gel, petroleum/acetone 3:1), yielding *R*-3-hydroxydecanoic acid (2.3 mg; identified by ^1^H-NMR), ^1^H-NMR (400 MHz, CD_3_OD): 3.96–3.98 (1H, *m*, H-3), 2.44 (1H, *dd*, *J* = 5.0, 15.5 Hz, H-2a), 2.36 (1H, *dd*, *J* = 8.1, 15.0 Hz, H-2b), 1.23–1.48 (14H, *m*, H-4, 5, 6, 7, 8, 9, 10), 0.90 (3H, *d*, *J* = 7.0 Hz, H-11).

### 3.5. Antibacterial Activity

Compound **1** was tested for antibacterial activity against *S*. *aureus* and MRSA strains (obtained from Food and Drug Administration of Hainan Province, Haikou, China) using the filter paper disc agar diffusion method [12]. The strains were cultured using nutrient agar. Fifty microliters (28 mM) of the compound were impregnated on sterile filter paper discs (6-mm diameter), and then, aseptically applied to the surface of the agar plates. Ten microliters (0.08 mg/mL) of kanamycin sulfate were used as positive control. Then the diameters of inhibition zones were measured after 24 h incubation at room temperature. Experiments were done in triplicate, and the results presented as mean values of the three measurements.

## 4. Conclusions

In summary, one new secondary metabolite was characterized from a marine-derived fungus A1 isolated from mangrove plant *S. hydrophyllacea* and its chemical structure was solved by spectroscopic and chemical analysis. Antibacterial tests demonstrated that compound **1** showed modest inhibitory effects on *Staphylococcus aureus* and methicillin-resistant *S*. *aureus* (MRSA), diameters of inhibition zones of which were 9.8 and 10.7 mm, respectively.

## Supplementary Files

Supplementary File 1: PDF-Document (PDF, 31 KB)
