# Supplementary material for: A Fatty Acid Glycoside from a Marine-Derived Fungus Isolated from Mangrove Plant Scyphiphora hydrophyllacea
Source: Mar Drugs. 2012 Mar 6;10(3):598–603. doi: 10.3390/md10030598 (PMC3347017; doi:10.3390/md10030598)
Supplement: Supplementary File 1: — PDF-Document (PDF, 31 KB) [file marinedrugs-10-00598-s001.pdf]

Supplementary Information

Figure 1S. Chromatograms of compound **1** and the extract of fungus A1.

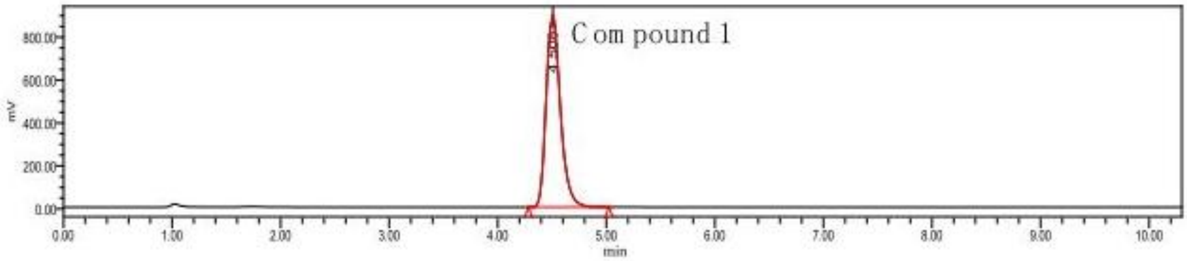

Name	Retention time	Area	% Area	Height	Integral type	Content	Unit	Peak type
1	4.507768	8170079	100	890623	BB			Unknown

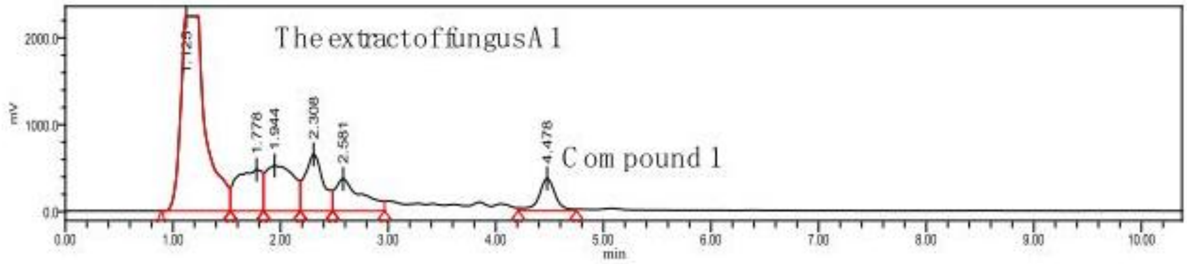

Name	Retention time	Area	% Area	Height	Integral type	Content	Unit	Peak type
6	4.477644	3970578	5.61409	371664	VV			Unknown
